# The dynamics of spatio-temporal Rho GTPase signaling: formation of signaling patterns

**DOI:** 10.12688/f1000research.7370.1

**Published:** 2016-04-26

**Authors:** Rafael Dominik Fritz, Olivier Pertz

**Affiliations:** 1 Department of Biomedicine, University of Basel, Basel, Switzerland; 2 Institute of Cell Biology, University of Bern, Bern, Switzerland

**Keywords:** Rho GTPase, Guanine nucleotide exchange factors, GTPase-activating proteins, spatio-temporal control, signalling patterns

## Abstract

Rho GTPases are crucial signaling molecules that regulate a plethora of biological functions. Traditional biochemical, cell biological, and genetic approaches have founded the basis of Rho GTPase biology. The development of biosensors then allowed measuring Rho GTPase activity with unprecedented spatio-temporal resolution. This revealed that Rho GTPase activity fluctuates on time and length scales of tens of seconds and micrometers, respectively. In this review, we describe Rho GTPase activity patterns observed in different cell systems. We then discuss the growing body of evidence that upstream regulators such as guanine nucleotide exchange factors and GTPase-activating proteins shape these patterns by precisely controlling the spatio-temporal flux of Rho GTPase activity. Finally, we comment on additional mechanisms that might feed into the regulation of these signaling patterns and on novel technologies required to dissect this spatio-temporal complexity.

## Introduction

Since the seminal articles from Allan Hall’s lab back in the early 1990s ^1– 3^, we have learned much about the biology of Rho GTPases ^4– 9^. The combination of experimental approaches, including genetics in model organisms, cell biology, and biochemistry, was key to establish the basics of Rho GTPase signaling. These techniques revealed the principles of GTPase regulation by guanine nucleotide exchange factors (GEFs), GTPase-activating proteins (GAPs), and Rho guanine nucleotide dissociation inhibitors (RhoGDIs), and identified effectors that exert specific biological functions downstream of Rho GTPases. This uncovered a surprisingly intertwined network of mutual regulatory protein complexes in which Rho GTPases are vastly outnumbered by GEFs, GAPs, and effectors ^10^.

In the last 15 years, an additional layer of complexity was superimposed on Rho GTPase biology. Fluorescence resonance energy transfer (FRET)-based and other biosensors enabled investigators to capture the spatio-temporal dimensions of Rho GTPase signaling in living cells with unprecedented resolution ^10, 11^. Visualizing Rho GTPase activity drastically changed our perception of Rho GTPase signaling and implies a higher degree of complexity than the classic ON-OFF schemes typically depicted in feed-forward, linear signaling networks. This fresh view emphasizes the importance of analyzing Rho GTPase activity dynamics by microscopy instead of analyzing steady states of limited information content by biochemistry.

Understanding that Rho GTPase signaling is organized in spatio-temporal patterns poses important novel questions: How are these signaling activity patterns generated? What forms their structural basis? And which technologies do we need to dissect the mechanisms of pattern formation in the future? In this review, we will survey the spatio-temporal activity patterns that have been documented to date and highlight possible answers to these intriguing questions. Then we discuss important players that might feed into this spatio-temporal regulation, and we comment on novel technologies to analyze the latter.

## Biosensors visualize Rho GTPase activity domains in time and space

**Figure 1.**
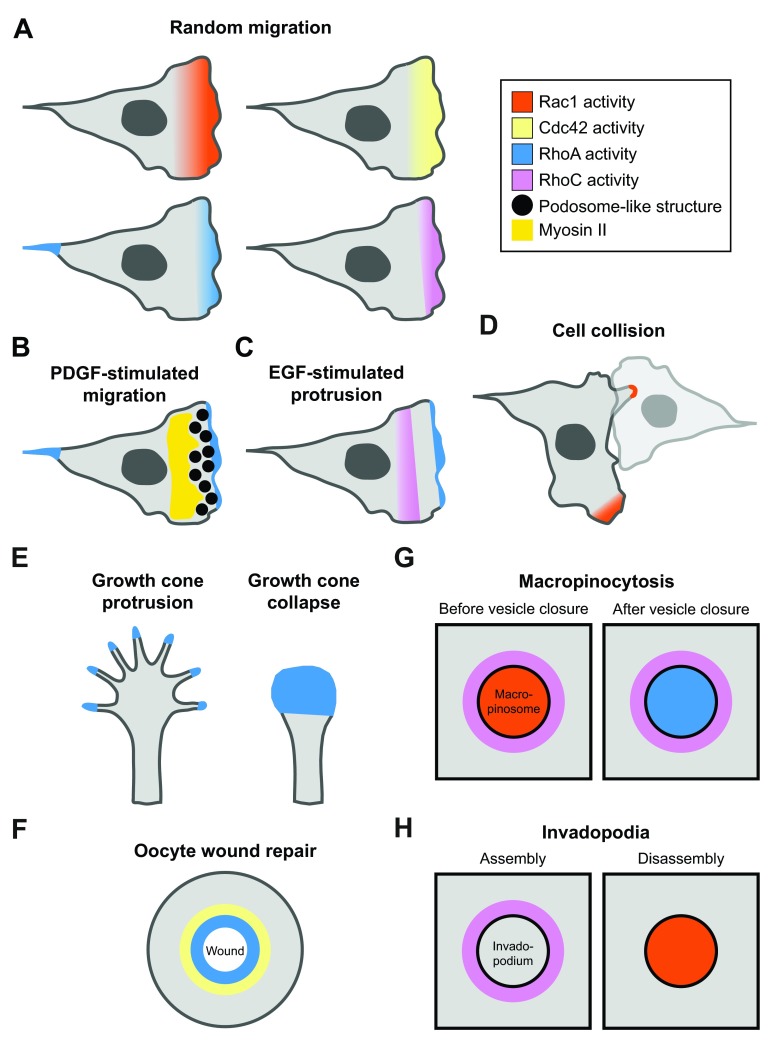
Spatial Rho-GTPase patterns in various cell systems. ( **A**) Rho GTPase activity gradients in randomly migrating fibroblasts. The activity is highest at the cell edge and declines toward the cell center. Color code is displayed to the right. ( **B**, **C**) Reshaping of Rho GTPase activity zones in response to growth factor treatment in fibroblasts ( **B**) or MTLN3 epithelial cells ( **C**). ( **D**) Rac1 activity in colliding fibroblasts. Rac1 is activated in a broad gradient in the contact-free protrusion (bottom) but restricted to a sharp band at the tip of the contact protrusion (top). ( **E**) RhoA activity at the tip of filopodia during growth cone protrusion and in the entire growth cone during collapse. ( **F**) Concentric Rho GTPase signaling zones during wound closure in *Xenopus* oocytes. ( **G**) Distinct Rho GTPase signaling domains in macropinocytosis. ( **H**) Rho GTPase activity domains during invadopodia assembly and disassembly. EGF, epidermal growth factor; PDGF, platelet-derived growth factor.

The traditional model of Rho GTPase signaling during cell migration states that Rac and Cdc42, respectively, regulate membrane protrusion and filopodia formation at the leading edge, whereas RhoA controls contractility at the trailing edge ^12^. However, the use of FRET biosensors proved this view to be too simplistic. Accordingly, Rac1, Cdc42, RhoA, and RhoC activity has been found at the leading edge in randomly migrating fibroblasts ( Figure 1A). While Rac1 forms a broad activity gradient that spans several micrometers into the cell interior ^13– 18^, Cdc42 ^14, 16, 19, 20^, RhoA ^16, 21– 24^, and RhoC ^24^ activity zones are somewhat narrower. Despite overlapping activity zones, the dynamics of all four Rho GTPases precisely correlate with cell edge protrusion/retraction dynamics ^16, 24, 25^. RhoA is also activated at the trailing edge during retraction and this suggests that RhoA is regulated by different GEFs, GAPs, and couples to distinct effectors to regulate edge dynamics or tail retraction ^10^.

The RhoA activity pattern considerably changes if fibroblasts are stimulated with platelet-derived growth factor (PDGF). On timescales of 10 to 20 minutes, PDGF stimulation leads to increased edge protrusion that correlates with immediate decrease of RhoA activity ^23, 25^. On a timescale of hours, homogeneous application of PDGF locks fibroblasts in a permanent state of persistent migration in one direction ^26^. This depends on the formation of podosome-like structures (PLSs), which broadly inhibits RhoA at the leading edge and simultaneously restricts RhoA activity to a sharp zone at the lamellipodium tip ( Figure 1B). Here, the PLSs function as a spatially organizing cytoskeletal module that defines the zones of high and low RhoA activity. This spatial organization of RhoA signaling uncouples myosin-based, actin retrograde flow from the leading edge, which is essential to maintain a polarized state required for persistent migration. Importantly, RhoA activity remains present at the back of PDGF-treated cells during tail retraction, further underpinning the local nature of spatio-temporal Rho GTPase regulation in different subcellular regions. Similarly, in epithelial cancer cells, epidermal growth factor (EGF) confines RhoA activity to the very edge of the cell and additionally shifts the diffuse RhoC activity pattern some micrometers back behind the edge in motile cells ^27^ ( Figure 1C). This is thought to position distinct effector pathways to coordinate leading-edge dynamics. These examples illustrate that activity patterns of particular Rho GTPases are highly dependent on the cellular context (that is, presence or absence of a growth factor, morphodynamic behavior such as edge protrusion and tail retraction, and cell type).

The plasticity of such spatio-temporal activity patterns was further demonstrated in fibroblasts undergoing cell-cell collisions ^18^. Colliding cells have two types of protrusions: contact protrusions, which touch the neighbor cell, and contact-free protrusions. The two protrusion types fundamentally differ in edge dynamics, which correlate with distinct Rac1 activity patterns. As observed earlier ^13– 17^, Rac1 activity forms a broad gradient in contact-free protrusions. In marked contrast, Rac1 activity is constrained to a narrow band at the tip of contact protrusions ( Figure 1D). This activity pattern correlates with formation of a robust F-actin band that allows contact protrusions to efficiently squeeze below adjacent cells. Again, the precise cellular context (presence or absence of cell-cell contact) dictates the shape of the Rho GTPase activity zone.

Rho GTPase activity zones have also been reported in cellular processes different from cell migration. In growth cones of neuroblastoma cells, RhoA is activated either locally or globally depending on the morphodynamic process ^21^. During growth cone protrusion, RhoA activity is detectable at the tip of F-actin bundles forming filopodia, where it most likely couples to the effector formin mDia to drive actin polymerization ( Figure 1E). In contrast, the collapsing growth cone displays bulk RhoA activity all over the retracting structure. Here, RhoA might interact with its effector Rho kinase to stimulate global actomyosin contractility.

The *Xenopus* oocyte wound repair process is another intriguing example of Rho GTPase activity patterning as it features two adjacent activity zones ( Figure 1F). Wounding rapidly activates both RhoA and Cdc42 that form local mutual exclusive activity rings that encircle the wound. The RhoA and Cdc42 zones colocalize with ring-like arrays of myosin-2 and F-actin, respectively, and coordinate the spatial regulation of both cytoskeletal structures to close the actomyosin ring inward and to seal the wound ^28, 29^.

Further concentric Rho GTPase activity zones were also found during macropinocytosis and the formation of invadopodia. In both cases, active RhoC surrounds macropinosomes ^24^ and invadopodia ^30^, and additional Rho GTPases are active in the core of these structures ( Figure 1G, H). RhoC is active during the entire macropinocytotic process ^24^, whereas Rac1 ^31^ and RhoA ^23^ activities peak before and after vesicle closure, respectively ( Figure 1G). Similar activity separation can be observed in invadopodia. Here, concentric RhoC activity drives invadopodia assembly ^30^, whereas Rac1 activity in the invadopodium’s core promotes its disassembly ^17^ ( Figure 1H).

In summary, multiple Rho GTPase activities can either overlap in time and space or form distinct zones, which are subject to modulation by growth factors and cell-cell interactions. Thus, rather than the classic dogma in which one Rho GTPase regulates one specific cytoskeletal structure, multiple Rho GTPases collaborate to fine-tune cytoskeletal dynamics at a specific subcellular location. The Rho GTPase activity zones then precisely position and coordinate multiple cytoskeletal regulating activities in time and space.

## GEF/GAP-mediated Rho GTPase fluxes underlie spatio-temporal signaling patterns

**Figure 2.**
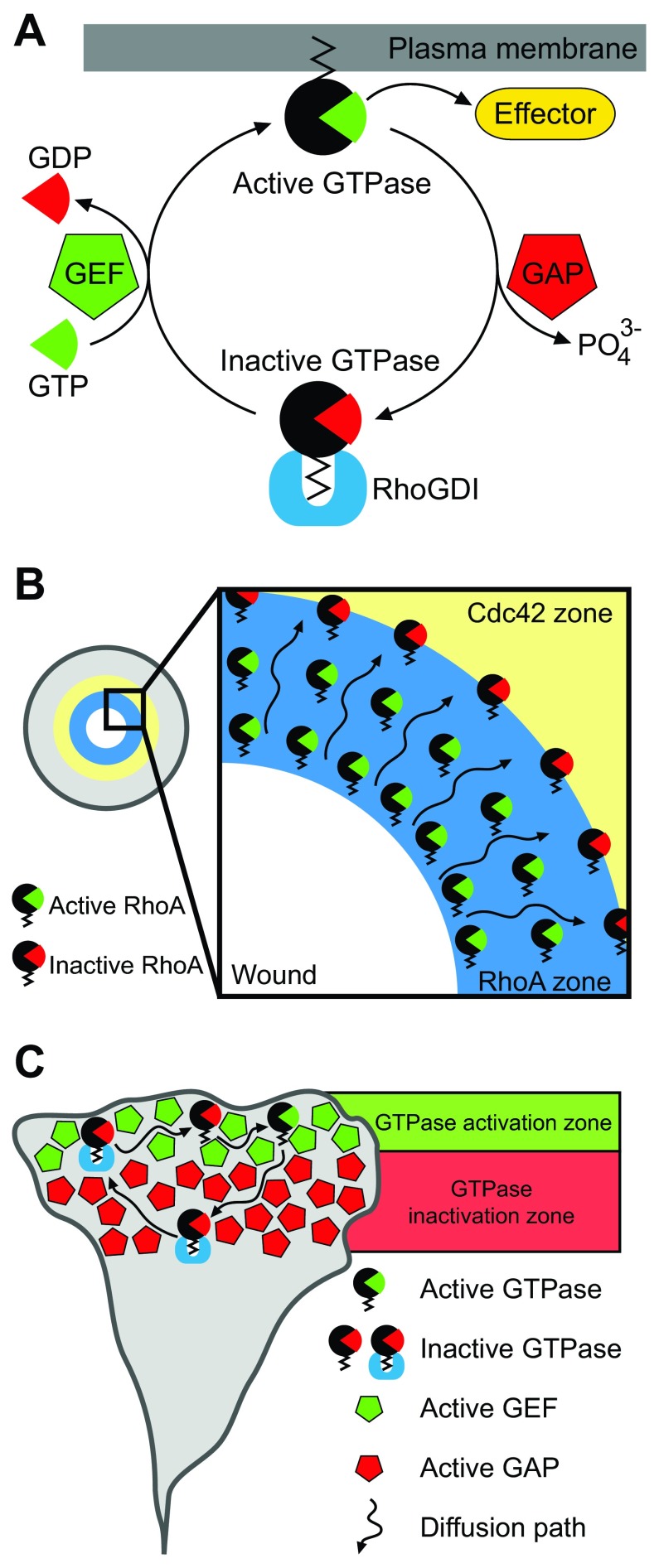
The Rho GTPase activation/deactivation cycle. ( **A**) Rho GTPases are kept in the cytoplasm by RhoGDIs. Activation occurs through GEF-mediated GTP loading and insertion of the GTPase into the membrane, where it interacts with downstream effectors. GAPs stimulate GTP hydrolysis to inactivate the Rho GTPase, which is sequestered in the cytoplasm by RhoGDI. ( **B**) RhoA signaling treadmill during oocyte wound closure. RhoA activation and inactivation occurs at opposite boundaries of the activity zone. RhoGDI is omitted for clarity. ( **C**) Possible view of the Rho GTPase lifecycle as a reaction-diffusion system. Spatial subcellular separation of GEFs and GAPs may determine distinct activation/deactivation zones, which maintain the Rho GTPase activity flux. GAP, GTPase-activating protein; GDP, guanosine diphosphate; GEF, guanosine nucleotide exchange factor; GTP, guanosine triphosphate.

An important question that immediately comes up is how these sharp or diffuse Rho GTPase activity zones are created. A possible answer to this fundamental question comes from the Rho GTPase life cycle ( Figure 2A). Rho GTPases are molecular switches that alternate between the active, GTP-loaded and inactive, GDP-loaded states. GEFs exchange GDP to GTP, whereas GAPs stimulate the hydrolysis of GTP to GDP. Additionally, active GTP-loaded GTPases reside in the membrane compartment where they interact with effector proteins. Conversely, inactive, GDP-loaded Rho GTPases are sequestered in the cytoplasm by RhoGDI. It has been proposed that this Rho GTPase cycling enables the dynamic signaling fluxes that are required to build spatially restricted signaling patterns. This has been mostly explored in the *Xenopus* egg wounding model system ^32^. As described above, oocyte wounding induces RhoA and Cdc42 activation within 20 seconds. At first, RhoA and Cdc42 activities form shallow and overlapping gradients that become steeper and eventually establish distinct concentric zones 90 seconds after wounding ^28^. Interestingly, both Rho GTPases cycle more rapidly between GTP- and GDP-loaded states inside activity zones than outside ^33^. Moreover, RhoA becomes preferentially inactivated at the trailing edge of the zone (that is, more distal with respect to the wound center), showing that a signaling treadmill generates a GTPase activity flux by proximal RhoA activation and distal RhoA inactivation within the zone ( Figure 2B). Experimental work and mathematical modeling further showed that the RhoA and Cdc42 concentric zones are partially shaped by the dual GEF-GAP Abr ^34, 35^. Abr is a GEF for RhoA, Rac, and Cdc42 and concomitantly a GAP for Rac and Cdc42 ^36^. Abr docks on active RhoA to generate a positive feedback loop that impinges on RhoA itself and simultaneously inhibits Cdc42 in the RhoA zone ^34, 35^. These data indicate that GEFs and GAPs regulate reaction-diffusion-based signaling fluxes that shape Rho GTPase activity zones during oocyte wound closure.

Similar mechanisms have been documented in mammalian cells. In the case of invadopodia formation, RhoC activity is spatially restricted in a concentric zone surrounding the invadopodium core through the interplay of p190RhoGEF and p190RhoGAP ^30^. Outside the core, p190RhoGEF activates RhoC, while p190RhoGAP localizes to the inner of the core where it inhibits RhoC. Another example is the regulation of the exquisitely focused RhoA activity pattern at the tip of F-actin bundles that form neuronal growth cone filopodia ( Figure 1D). A recent study identified the RhoA-specific GAP DLC1 (deleted in liver cancer 1) to spatially regulate the filopodial RhoA activity pattern ^37^. RNA interference (RNAi)-mediated DLC1 knockdown leads to widening of the RhoA activity domain, suggesting that DLC1 acts by shaping the focused RhoA activity zone at filopodial tips. Together, these data clearly suggest that fine spatial regulation of Rho GTPase activation/deactivation cycles enables the formation of a signaling pattern.

Taking into consideration the aforementioned examples, we propose a general mechanism of Rho GTPase pattern formation based on reaction-diffusion systems. Such a Rho GTPase activity pattern would be dynamically maintained by successive cycles of (1) local activation by a GEF, (2) slow plasma membrane (PM) diffusion (0.02 to 1.36 µm ^2^ s ^−1^) ^38, 39^ from a zone preferentially occupied by a GEF to a zone preferentially occupied by a GAP, (3) local inactivation by the GAP, and (4) membrane extraction by RhoGDI. Once in the cytoplasm, the Rho GTPase-RhoGDI complex can quickly diffuse (10 to 100 µm ^2^ s ^−1^) ^40^ and reach an equilibrium within the cytosol before being used for subsequent activation cycles ( Figure 2C). Such a constant reaction-diffusion system requires spatially regulated GEFs and GAPs. Additionally, regulation of membrane/cytosol partitioning by RhoGDI will most likely also feed into the shaping of spatio-temporal Rho GTPase activity patterns. Membrane/cytosol partitioning is subject to modulation by multiple protein kinases, which determine the release of specific Rho GTPases from the cytosolic RhoGDI-bound pool or the affinity of Rho GTPases for membranes (reviewed in 5). The impact of RhoGDI on Rho GTPase activity pattern formation is underpinned by the comparison of RhoGDI-responsive and non-responsive FRET sensors ^21^. In the case of RhoA, a biosensor version that does not bind to RhoGDI and thus is constitutively targeted to the PM shows global activation in the neuronal growth cone. In contrast, a biosensor that retains the ability to bind to RhoGDI displays the highly focused filopodial RhoA activity pattern described above ( Figure 1E). It is therefore important to consider that constitutively membrane-bound Rho GTPase FRET biosensors might miss some aspects of spatio-temporal Rho GTPase signaling.

Only a few examples of spatio-temporal Rho GTPase regulation mechanisms by GEFs and GAPs have been studied up to now. An important question that emerges from the initial data we have discussed above is how GEFs and GAPs are themselves spatially regulated. Below, we review a large number of possible GEF/GAP interactions that might feed into this spatio-temporal regulation. This provides an idea of the players and mechanisms that will have to be studied to understand spatio-temporal Rho GTPase regulation.

## Spatio-temporal regulation of GEFs and GAPs

### Membrane composition and topology

**Figure 3.**
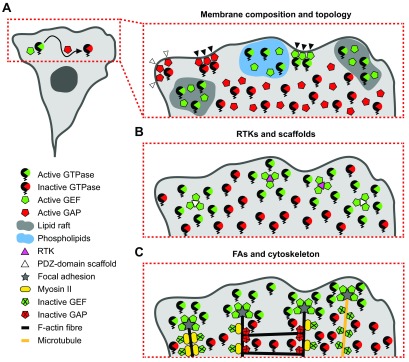
Potential GEF/GAP positioning mechanisms to create local Rho GTPase activity zones. ( **A**) Membrane composition and topology influence the recruitment of GEFs and GAPs to the PM. Lipid rafts and phospholipid-enriched zones attract GEFs and facilitate Rho GTPase activation. Certain GAPs can localize to protruding convex cell edges (white arrowheads), while concave cell edges can recruit both GEFs and GAPs (black arrowheads). The key to the left indicates the various symbols. RhoGDI is omitted for clarity. ( **B**) Receptor tyrosine kinases (RTKs) and PDZ-domain scaffold proteins cluster GEFs and might create local Rho GTPase activation zones. ( **C**) Focal adhesions (FAs) serve as GEF enrichment structures and locally activate Rho GTPase signaling. F-actin and microtubules sequester and inactivate GEFs and GAPs that become active upon release into the cytosol. GAP, GTPase-activating protein; GEF, guanosine nucleotide exchange factor; PDZ, PSD95-Dlg1-ZO1; PM, plasma membrane.

Almost all GEFs bear a lipid-interaction domain ^37, 39, 40^: a pleckstrin homology (PH), a DOCK homology region 1 (DHR-1), or a Bin-Amphiphysin-Rvs (BAR) domain ^41– 43^. Many GAPs also contain a variety of lipid-binding domains ^44^. Since PH and DHR-1 domains vary in their binding specificity and affinity for phospholipids such as phosphatidylinositol 4,5-bisphosphate (PIP _2_) and phosphatidylinositol (3-5)-triphosphate ^42, 45, 46^, GEFs and GAPs might be directed to specific PM subdomains ( Figure 3A). Indeed, distinct lipid distributions were found in the RhoA and Cdc42 zones during oocyte wound closure ^47^. Furthermore, specific sorting has been shown for the Rac GAP β2-chimaerin that localizes and inhibits Rac in the non-lipid raft zone ^48^ and p190 RhoGAP, which translocates to lipid rafts to cease RhoA activity in response to growth factor treatment ^49^. BAR domains recognize membrane curvature and thus target proteins to specific PM topologies ^50^. One GEF and seven GAPs with different BAR domains have been identified to date ^51, 52^. The F-BAR domain of srGAP2 was recently shown to tether the Rac GAP exclusively to convex, protruding membranes where it limits the duration of Rac1 activity during cell-cell collision without affecting the shape of the Rac1 activity pattern *per se* ^18^. Since srGAP2 integrates both membrane topology and Slit-Robo repulsive signals, this mechanism ensures that srGAP2 inactivates Rac1 at the right subcellular region and in a specific morphodynamic phase.

Besides mere targeting of GAPs, lipids also influence both GAP activity and the specificity toward particular Rho GTPases *in vitro*. The Rac- and Cdc42-specifc GAP n-Chimaerin is inhibited by phosphatidylserine and phosphatidic acid but activated by PIP _2_ and arachidonic acid ^53^. Some phospholipids have also been reported to switch the specificity of p190RhoGAP by inhibiting its GAP activity for Rho and stimulating its activity for Rac1 ^54^. Since lipid distribution can be highly ordered in the PM, these results strongly suggest that both lipid species and membrane topology can create Rho GTPase signaling microdomains.

### Interaction with receptor tyrosine kinases and scaffold proteins

Receptor tyrosine kinases (RTKs) play a paramount role in Rho GTPase activation ^55^ and are very likely to determine their spatio-temporal activity in two ways. First, RTKs alter the lipid composition of the PM through activation of phosphatidylinositol-3 kinase and phospholipase Cγ ^56^ to influence GEF and GAP targeting as described above. Second, RTKs recruit GEFs directly and activate them by phosphorylation ( Figure 3B). For instance, Tiam1 ^57^, LARG ^58^, Vav1-3 ^59– 62^, Vsm ^63^, Dbs ^64^, RasGfr1 ^65^, and Kalirin ^66^ are found in complexes with various RTKs and some of them also become activated by phosphorylation ^59, 60, 62– 65, 67, 68^. Since RTKs themselves are capable of generating spatial signaling patterns at the level of their phosphorylation ^69^, this might serve as an additional way to spatio-temporally regulate Rho GTPases. G protein-coupled receptors have also been shown to feed into the regulation of Rho GTPase signaling ^70^, but their contribution to spatio-temporal regulation has not yet been explored.

A striking feature in approximately 40% of GEFs is the presence of a PSD95-Dlg1-ZO1 (PDZ) domain-binding motif at the C-terminus. These GEFs interact with various scaffold proteins such as Shank and Scribble ^71^. Shank positions β-Pix in the postsynaptic density region to locally control Rac1-dependent dendritic spine formation ^72, 73^, whereas Scribble recruits β-Pix to the PM to regulate thyrotropin receptor trafficking ^74, 75^. Interestingly, the Scribble/β-Pix interaction is modulated by TIP-1, which competes for β-Pix binding and affects its subcellular localization ^76^.

### Adhesion complexes and the cytoskeleton

Adhesion complexes are further important hubs for spatial regulation of GEFs and GAPs ( Figure 3C). β-Pix is directly recruited from the cytosol to focal adhesions (FAs) at the leading edge of migrating cells through the interaction with the Paxillin-GIT-PAK complex ^39, 77^. At adhesions, focal adhesion kinase (FAK) phosphorylates β-Pix to strengthen the β-Pix-Rac1 interaction and thus enhance Rac1 recruitment to adhesions ^78, 79^. Notably, the balance of β-Pix distribution between FAs and endosomes is regulated by the PDZ domain-containing sorting nexin 27 ^80^. Another GEF that activates Rac at FAs is DOCK180. Its polarized localization to the leading edge is mediated through interaction with the Paxillin-p130Cas-CrkII complex following integrin engagement ^81– 83^. Furthermore, the Rac GEF Tiam1 was found to bind to talin in FAs and to activate Rac1 in a PAR complex-dependent manner ^84^. There are also several Rho-specific GEFs enriched at FAs in a FAK-dependent manner. Net1 is present in FA complexes at the leading and the trailing edge ^85^, whereas PDZ-RhoGEF localizes to the trailing edge only ^86, 87^. LARG and p115RhoGEF also interact with FAK at adhesions ^86, 88^. These four Rho-specific GEFs seem to regulate similar processes, although it is not fully understood yet whether their functions are redundant or whether they depend on different upstream signals.

The cytoskeleton also tethers GEFs and GAPs. Active myosin II (MII), which generates actomyosin-based contractility, sequesters and inactivates β-Pix at actin fibers and thus confers an MII- and β-Pix-dependent front-back polarity in migrating cells ^89, 90^. Thus, MII orchestrates adhesion formation and maturation by adsorption and release of β-Pix. Notably, MII sequesters and inhibits GEFs containing a Dbl homology domain such as FGD1, Kalirin, LARG, DOCK180, Tiam1, Trio, GEF-H1, and Dbl ^89^. F-actin also traps GAPs as FilGAP, which binds to the F-actin cross-linker filamin A. After mechanical deformation of F-actin branches, FilGAP is released and translocates to the PM to inhibit Rac at the leading edge ^91^. Finally, microtubules tie and inactivate GEF-H1, which is released after the depolymerization of microtubules to stimulate RhoA activity and contractility at the leading edge ^92, 93^.

In summary, growth factors, mechanosensation, membrane topology/composition, and the actomyosin and tubulin cytoskeletons regulate the spatio-temporal aspects of Rho GTPase activity patterns, which in turn feedback on these different organizational and signaling levels. It is now time to explore how this plethora of different GEF/GAP regulatory mechanisms impact on spatio-temporal Rho GTPase activation.

## Conclusions

The technological progress in the last 15 years has empowered us with the ability to monitor Rho GTPase signaling with high spatio-temporal resolution. With respect to initial models, this has revealed an unexpected spatio-temporal signaling complexity, which now needs to be systematically analyzed by perturbation of the different players we have discussed in this review. Because spatio-temporal Rho GTPase signaling patterns are constantly regulated on timescales of tens of seconds, novel technologies are required to perturb cell systems at that exact timescale. This can take advantage of existing techniques such as optogenetics or small-molecule dimerizers to control GEF/GAP targeting and activity ^94– 98^. Dissection of the complexity of spatio-temporal Rho GTPase signaling patterns will also require obtaining biophysical parameters with subcellular resolution, which can for example be inferred from fluorescence correlation spectroscopy ^99^. Ultimately, such multidisciplinary approaches will inform mathematical models that can describe the network properties required to generate robust spatial signaling patterns ^100^. In addition to *in vitro* experiments, analyzing Rho GTPase activity by FRET reporters *in vivo* ^101^ will guarantee new insights, provided that RhoGDI-responsive sensors are used. Beyond the goal of understanding how Rho GTPase signaling is spatio-temporally regulated, these approaches will also unveil how Rho GTPase coordinately regulate different cytoskeletal polymers to fine-tune the highly complex and dynamic processes required for cell morphogenesis. We foresee that a limited number of conserved spatio-temporal Rho GTPase networks will emerge from systematic perturbation approaches. Tuning of a limited number of parameters might then allow the cell to repurpose such networks to regulate edge protrusion, growth cone motility, macropinocytosis, sealing of a cell wound, or other morphodynamic processes.

## Abbreviations

BAR, Bin-Amphiphysin-Rvs; DHR-1, DOCK homology region 1; FA, focal adhesion; F-actin, filamentous actin; FAK, focal adhesion kinase; FRET, fluorescence resonance energy transfer; GAP, GTPase-activating protein; GEF, guanosine triphosphate exchange factor; GTP, guanosine triphosphate; PAK, p21-activated kinase; PDGF, platelet-derived growth factor; PDZ, PSD95-Dlg1-ZO1; PH, pleckstrin homology; PIP _2_, phosphatidylinositol 4,5-bisphosphate; PLS, podosome-like structure; PM, plasma membrane; RhoGDI, Rho guanine nucleotide dissociation inhibitor; RTK, receptor tyrosine kinase.
